# Charge transport in DNA model with vibrational and rotational coupling motions

**DOI:** 10.1007/s10867-017-9455-6

**Published:** 2017-07-20

**Authors:** H. Ngoubi, G. H. Ben-Bolie, T. C. Kofané

**Affiliations:** 1 Laboratory of Biophysics, Department of Physics, Faculty of Science, University of Yaounde I, P.O. Box 812, Yaounde, Cameroon; 2 Laboratory of Nuclear Physics, Department of Physics, Faculty of Science, University of Yaounde I, P.O. Box 812, Yaounde, Cameroon; 3 Laboratory of Mechanics, Department of Physics, Faculty of Science, University of Yaounde I, P.O. Box 812, Yaounde, Cameroon

**Keywords:** Charge transport, Peyrard-Bishop model, Transfer constant, Size of nucleotide

## Abstract

The dynamics of the Peyrard-Bishop model for vibrational motion of DNA dynamics, which has been extended by taking into account the rotational motion for the nucleotides (Silva et al., J. Biol. Phys. **34**, 511–519, 2018) is studied. We report on the presence of the modulational instability (MI) of a plane wave for charge migration in DNA and the generation of soliton-like excitations in DNA nucleotides. We show that the original differential-difference equation for the DNA dynamics can be reduced in the continuum approximation to a set of three coupled nonlinear equations. The linear stability analysis of continuous wave solutions of the coupled systems is performed and the growth rate of instability is found numerically. Numerical simulations show the validity of the analytical approach with the generation of wave packets provided that the wave numbers fall in the instability domain.

## Introduction

The development of the theory of the modulation instability (MI) as a well-known nonlinear phenomenon has attracted considerable attention and started almost simultaneously and occurred in parallel in hydrodynamics by Benjamin and Feir [1, 2] and in electrodynamics by Ostrovskii et al. [3, 4]. It can be found in many fields of physics, such as fluid dynamics [5], nonlinear optics. MI, an effect well known in the latter context, is the process by which a constant-wave background becomes unstable to sinusoidal modulations because of the presence of a focusing nonlinearity, leading to a pulsed wave, called a bright soliton train [6, 7]. MI in optical fibers was originally studied by Hasegawa and Brinkman [6] and their theoretical prediction was observed experimentally by Tai et al. [7].

Nonlinear excitations [8–19] (solitons, discrete breathers, intrinsic localized modes) have been drawing increasing attention over recent years and are widely believed to be responsible for several effects in molecular chains, such as charge and thermal conductivity, energy transfer and localization. A particularly interesting discrete system that support solitons and localized modes is deoxyribonucleic acid, or DNA. In this system, localization of energy has been suggested as a precursor of the transcription bubble [10], and moving localized oscillations as a method of transport of information along the double strand [15]. For this reason Fialko and Lakhno have studied the transport of charge and hole along the short DNA molecule by using the Peyrard-Bishop-Holstein (PBH) model [20, 21]. These authors investigate the impact of long-range transfer of charges through the DNA molecule. Many authors have shown that the most standard mechanism through which bright solitons or solitary wave structures appear is through the activation of MI of plane waves. In the above-mentioned contexts, MI has been suggested to be responsible for energy localization mechanisms leading to the formation of large amplitude nonlinear excitations in hydrogen-bonded crystals or DNA molecules.

Peyrard and Bishop [10] studied the process of denaturation in which only the transverse motion of bases along the hydrogen bond was taken into account. One of its improved version, proposed by Dauxois et al. [8], takes helicity into consideration and has been recently shown, by Zdravkovic et al. [11, 12] and then by Tabi et al. [13, 14], to better describe the extremely high amplitude stretching of the hydrogen bonds. It is therefore obvious that all the above-cited models describe either the radial opening of strands or the unwinding of the double helix, but not both of them simultaneously. In more recent years, due to experiments on single molecules of DNA, models with two and more degrees of freedom have been introduced with emphasis on the radial and torsional aspects. As an example, a model which has two degrees of freedom per base pair: one radial variable related to the opening of the hydrogen bonds and an angular one related to the twisting of each base-pair responsible for the helicoidal structure of the molecule, has been built by Barbi et al. [15], and later improved by Cocco and Monasson [16]. Such a model provides an extension of the Peyrard-Bishop approach towards a more realistic description of biological processes. It can also be useful for more general studies of the interaction between geometrical conformation and dynamical properties of the molecule referring to the recent mechanical experiments on DNA [17]. Nonlinear interactions between atoms in DNA can give rise to intrinsically localized breather-like vibration modes [18]. Such localized modes, being large amplitude vibrations of a few (2 or 3) particles, can facilitate the disruption of base pairs and in this way initiate conformational transitions in DNA. These modes can occur as a result of the modulational instability of continuum-like nonlinear modes [19], which is created by energy exchange mechanisms between the nonlinear excitations. The latter favors the growth of large excitations [22]. But, as far as we know, no work concerning analysis of MI through the Peyrard-Bishop model of DNA with rotational motion for the nucleotides has been presented in the literature. So, the aim of the present work is to show that the nonlinear dynamics of DNA can be described by coupled discrete equations. The paper is organized as follows. In Section 2, we propose the model and we derive the equations of motion for exciton and phonon excitations. In Section 3, linear analysis is investigated and predictions of some localized structure formations are made. The validity of this analysis is checked by numerical simulations in Section 4. Section 5 concludes the paper.

## Model and equations of motion

The Peyrard-Bishop (PB) model was introduced in 1989 to explain the fluctuations and the nonlinear phenomena observed in the DNA molecule [10]. Initially for this model, only one degree of freedom per base pair was recognized, the opening along the hydrogen bond of base pair *n*. Thereafter, the model with several degrees of freedom per base pair was elaborated by taking into account simultaneously the transverse and longitudinal motions characterized by *u*_*j*_ and *v*_*j*_ starting from the equilibrium position and the rotational motions characterized by *𝜃*_*j*_ and *ϕ*_*j*_ [23]. Each nucleotide is represented by a disc of mass *m*, the radius *r* and *d* represent in the equilibrium position between the discs. On each base pair, forces of interactions with the other base pair are exerted, which are represented by a nonlinear potential *V* (*u*_*j*_,*v*_*j*_,*𝜃*_*j*_,*ϕ*_*j*_) [23]. The displacement of the *H* bond between two adjacent discs *j* used in this present work, can be written as [23].

1 $$ y_{j}=(u_{j}-v_{j}+d+2r)-r(\cos\theta_{j}+\cos\phi_{j}). $$

The lattice Hamiltonian for the system can be written as [23]

2 $$\begin{array}{@{}rcl@{}} H_{1}&=&\sum\limits_{j}\left\{\frac{p_{w_{j}}^{2}}{2m}+\frac{p_{\lambda_{j}}^{2}}{2m} +\frac{p_{\phi_{j}}^{2}}{2I}+\frac{p_{\theta_{j}}^{2}}{2I}\right\}+ \sum\limits_{j}\left\{\frac{k}{2}\left[(w_{j}-w_{j-1})^{2}+(\lambda_{j}-\lambda_{j-1})^{2}\right]\right\}\\ &&+ \sum\limits_{j}\left\{\frac{\xi}{2}\left[(\phi_{j}-\phi_{j-1})^{2}+(\theta_{j}-\theta_{j-1})^{2}\right]\right\}+\sum\limits_{j} U(x_{j}) \end{array} $$

where

3 $$\begin{array}{@{}rcl@{}} w_{j}&=&\frac{u_{j}+v_{j}}{\sqrt{2}},\\ \lambda_{j}&=&\frac{u_{j}-v_{j}}{\sqrt{2}}, \end{array} $$

In this equation, $ p_{w_{j}}$ (or $p_{\lambda _{j}}$) and $ p_{\phi _{j}}$ (or $p_{\theta _{j}}$) are the linear and angular moments, respectively. We consider that the mass *m*, the moment of inertia *I* are equal for all nucleotides [23]. Also, the strength constants *k* and *ξ* are the same for the system. In this present work we consider only the artificial and the homogeneous DNA molecule. Consequently all base pairs are identical. All parameters used in the work have been taken from in the references [24–26]. The interaction between two adjacent bases is given by the Morse potential [25]

4 $$ U\left( {y_{j}} \right) = D\left[ {\exp \left( {- a(y_{j}-y_{0}) } \right) - 1} \right]^{2}= D\left[ {\exp \left( {- a\sqrt{2}\lambda_{j}-g(\phi_{j},\theta_{j}) } \right) - 1} \right]^{2} $$

where *D* is the energy of dissociation of the base pair, is a parameter with dimension of inverse length, *y*_0_ = 2*r* + *d* is the equilibrium point (distance between the centers of the discs), and the function *g*(*ϕ*_*j*_,*𝜃*_*j*_) is given by

5 $$ g(\phi_{j},\theta_{j})=r(\cos\phi_{j}+\cos\theta_{j}). $$

In the following, the above extended PB model will be coupled to the excitons. Thus, the Hamiltonian of excitons for the system can be written as

6 $$ H_{2} = - {\sum}_{j} {V\left( {B_{j}^ + B_{j + 1} + B_{j}^ + B_{j - 1} } \right)}. $$

This Hamiltonian describes the transfer of charges between the pairs of bases in the adjacent stacking of the molecular orbitals which superimpose along the DNA molecule. $B_{j}^{+}$ and *B*_*j*_ are creation and annihilation operators, respectively, for charge carrier at the *j* th base pair of the double strand, and *V* represents the coupling transfer integral between π orbital at adjacent base pairs. For the charge-lattice interaction, we use on-site, Holstein-type coupling [26, 27]. Thus, the Hamiltonian interaction of the system can be expressed as:

7 $$ H_{3} = {\sum}_{j} {\chi \lambda_{j} } B_{j}^ + B_{j}, $$

where *χ* is the coupling vibrational and rotational constant. The total Hamiltonian of our system can be written as *H* = *H*_1_ + *H*_2_ + *H*_3_. Now, we use semiclassical equations of motion [26], i.e, we treat the quantum charge mechanically and the vibrational and rotational motion classically. We obtain the following Schrödinger equation:

8 $$ i\hbar \frac{d}{dt}\varphi_{j} = -V\left( {\varphi_{j + 1} + \varphi_{j - 1} } \right) + \chi \lambda_{j} \varphi_{j} $$

where *φ*_*j*_ is the probability amplitude for the charge carrier located at the *j* th base pair. Newton’s equations of motion for the strectching *w*_*j*_, *λ*_*j*_, and the rotational *𝜃*_*j*_, *ϕ*_*j*_ motions become:

9 $$ m{ \ddot{w}_{j}}=k(w_{j+1}+w_{j-1}-2w_{j}), $$

10 $$\begin{array}{@{}rcl@{}} m\ddot{\lambda}_{j}&=&k(\lambda_{j+1}+\lambda_{j-1}-2\lambda_{j})+2\sqrt{2}aD(\exp(-a\sqrt{2}\lambda_{j}-g(\theta_{j}, \phi_{j}))-1)\\ &&\times (\exp(-a\sqrt{2}\lambda_{j}-g(\theta_{j},\phi_{j})))-\chi\lambda_{j}|\varphi_{j}|^{2}, \end{array} $$

11 $$\begin{array}{@{}rcl@{}} I\ddot{\phi}_{j}&=&\xi(\phi_{j+1}+\phi_{j-1}-2\phi_{j})-2raD\sin\phi_{j}(\exp(-a\sqrt{2}\lambda_{j}-g(\theta_{j}, \phi_{j}))-1)\\ &&\times (\exp(-a\sqrt{2}\lambda_{j}-g(\theta_{j},\phi_{j}))), \end{array} $$

12 $$\begin{array}{@{}rcl@{}} I\ddot{\theta}_{j}&=&\xi(\theta_{j+1}+\theta_{j-1}-2\theta_{j})-2raD\sin\theta_{j}(\exp(-a\sqrt{2}\lambda_{j}- g(\theta_{j},\phi_{j}))-1)\\ &&\times (\exp(-a\sqrt{2}\lambda_{j}-g(\theta_{j},\phi_{j}))). \end{array} $$

Equations (8)–(12) are equations of motion of our system. Let:

13 $$\begin{array}{@{}rcl@{}} X_{j}=\frac{\theta_{j}+\phi_{j}}{\sqrt{2}}, \\ {\Psi}_{j}=\frac{\theta_{j}-\phi_{j}}{\sqrt{2}}, \end{array} $$

14 $$\begin{array}{@{}rcl@{}} x_{j}=\frac{X_{j}}{\sqrt{2}},\\ \psi_{j}=\frac{{\Psi}_{j}}{\sqrt{2}}. \end{array} $$

Our priority is to transform (11)–(12) into one equation. Substituting (13)–(14) into (11)–(12), we obtain the following equation:

15 $$\begin{array}{@{}rcl@{}} I\ddot{\psi}_{j}&=&\xi(\psi_{j+1}+\psi_{j-1}-2\psi_{j})-2raD\sin\psi_{j}\cos x_{j}(\exp(-a\sqrt{2}\lambda_{j}-g(x_{j},\psi_{j}))-1)\\ &&\times(\exp(-a\sqrt{2}\lambda_{j}-g(x_{j},\psi_{j}))) \end{array} $$

where *g*(*X*_*j*_,Ψ_*j*_) = *g*(*x*_*j*_,*ψ*_*j*_) = 2*r*(cos*x*_*j*_ cos*ψ*_*j*_). Equation (9) represents a pure harmonic lattice with plane solutions. Our attention will be focused on the nonlinear (10)–(12). For small angular motion we have cos*x*_*j*_ ≃ 1, sin*ψ*_*j*_ ≃ *ψ*_*j*_ and $\exp (-a\sqrt {2}\lambda _{j}-g(x_{j},\psi _{j}))\simeq \exp (-a\sqrt {2}\lambda _{j})$ because *g*(*x*_*j*_,*ψ*_*j*_) ≃ 2*r* and exp(−2*r*) → 0 when *r* →*∞* Using this approximation, and (15) becomes:

16 $$ I\ddot{\psi}_{j}=\xi(\psi_{j+1}+\psi_{j-1}-2\psi_{j})-2raD\psi_{j}(\exp(-a\sqrt{2}\lambda_{j})-1) (\exp(-a\sqrt{2}\lambda_{j})). $$

Finally, after using all cited approximations, the equations of motion becomes three discrete equations for three variables,

17 $$ i\hbar \frac{d}{dt}\varphi_{j} = -V\left( {\varphi_{j + 1} + \varphi_{j - 1} } \right) + \chi \lambda_{j} \varphi_{j}, $$

18 $$ I\ddot{\psi}_{j}=\xi(\psi_{j+1}+\psi_{j-1}-2\psi_{j})-2raD\psi_{j}(\exp(-a\sqrt{2}\lambda_{j})-1) (\exp(-a\sqrt{2}\lambda_{j})), $$

19 $$\begin{array}{@{}rcl@{}} m\ddot{\lambda}_{j}&=&k(\lambda_{j+1}+\lambda_{j-1}-2\lambda_{j})+2\sqrt{2}aD(\exp(-a\sqrt{2}\lambda_{j}-g(\theta_{j},\phi_{j}))-1)\\ &&\times (\exp(-a\sqrt{2}\lambda_{j}-g(\theta_{j},\phi_{j})))-\chi\lambda_{j}|\varphi_{j}|^{2}. \end{array} $$

After expanding the terms in the exponential up to the third order and using the continuum approximation, we obtain the following equations

20 $$ \ddot{\lambda}+c_{0}\frac{\partial^{2}\lambda} {\partial x^{2}}+W_{g}(\lambda-\alpha_{0}\lambda^{2}+\gamma_{2}\lambda^{3})+c_{2}|\varphi|^{2}=0, $$

21 $$ \ddot{\psi}+c_{1}\frac{\partial^{2}\psi} {\partial x^{2}}-\beta(\psi\lambda-\alpha_{0}\psi\lambda^{2}+\gamma_{2}\psi\lambda^{3})=0, $$

22 $$ i\frac{d}{dt}\varphi= P_{1}\frac{\partial^{2}\varphi}{\partial x^{2}}+Q_{1}\varphi+Q_{2}\lambda\varphi, $$

where $W_{g}=\frac {4a^{2}D}{m}$, $\alpha _{0}=\frac {3a\sqrt {2}}{2}$, $c_{0}=\frac {-k}{m}$, $c_{1}=\frac {-\xi }{I}$, $\gamma _{2}=\frac {7a^{2}}{3}$, $\beta =\frac { m r W_{g}\sqrt {2}}{2I}$, $Q_{1}=-\frac {V}{\hbar }$, $Q_{2}=\frac {\chi }{\hbar }$, $P_{1}=-\frac {2V}{\hbar }$ and $c_{2}=\frac {\chi }{m}$.

## Linear stability analysis

In order to perform the linear stability analysis of system (20) and (21), we assume that:

23 $$\begin{array}{@{}rcl@{}} \varphi=\varphi_{0}\exp i(k x-w_{0}t),\\ \lambda=\lambda_{0} ,\\ \psi=\psi_{0}\exp i(k x-w_{0}t), \end{array} $$

with real constants *w*_0_, *λ*_0_, *ψ*_0_ and *φ*_0_ complex constant and where and are the wave number and frequency, respectively, of the system without perturbation. Introducing the above relation into (20) and (21), we get:

24 $$ w_{0}= \eta (\frac{A-k^{2}}{B+k^{2}}), $$

where

25 $$\begin{array}{@{}rcl@{}} A=\frac{\beta c_{2}}{W_{g} c_{1}}, \\ B=\frac{Q_{1}+Q_{2}\lambda_{0}}{P_{1}}, \\ \eta =\frac{c_{1}}{P_{1}} \end{array} $$

and

26 $$ k^{4}-\mu k^{2}+\mu_{1}=0 $$

where

27 $$ \mu=-\frac{1}{{P_{1}^{2}}}(2P_{1}(Q_{1}+Q_{2}\lambda_{0})+c_{1}) $$

28 $$ \mu_{1}=- \beta c_{2} \frac{|\varphi_{0}|^{2}}{W_{g}}+(Q_{1}+Q_{2}\lambda_{0})^{2}. $$

Equation (26) have solutions given by:

29 $$ k_{+}^{2}=\frac{\mu+(\sqrt{\mu^{2}-4\mu_{1})}}{2}, $$

30 $$ k_{-}^{2}=\frac{\mu-(\sqrt{\mu^{2}-4\mu_{1})}}{2}. $$

**Fig. 1:**
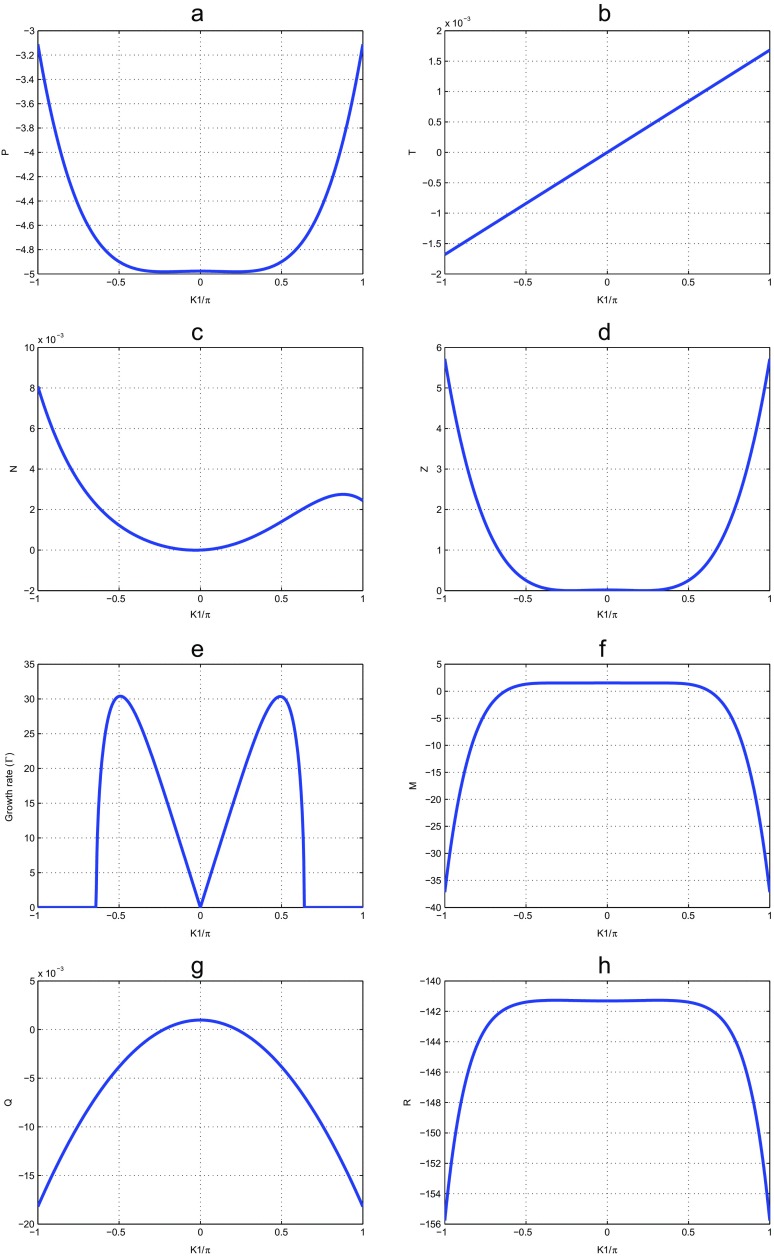
Parameters of the nonlinear dispersion relation (44) for *m* = 300*a* *m* *u*, *a* = 4.41Å ^−1^,*χ* = 1.2*e* *V*Å ^−1^,*D* = 0.05*e* *V*,*V* = 0.005*e* *V*,*k* = 0.04,*r* = 0.3Å ^−1^,*I* = 300*a* *m* *u*,*φ*_0_ = 10^−3^Å ^−1^

The major remark is that all the particles of the molecular chain oscillate in all the directions (see Fig. 1), creating nonlinear effects in the molecule. Figure 1 plots the coefficients of nonlinear dispersion relation versus the wave number *K*_1_ and we note different behaviors in the dynamics of the base pairs. We see that the dispersion curve oscillates. This behavior is probably induced by the competition between the longitudinal and the rotational motions. Adding a small perturbation in above the equilibrium state, that is

31 $$\begin{array}{@{}rcl@{}} \varphi=(\varphi_{0}+\epsilon\varphi_{1})\exp i(K x+w_{0}t),\\ \lambda=\lambda_{0}+\epsilon\lambda_{1},\\ \psi=(\psi_{0}+\epsilon\psi_{1})\exp i(K x+w_{0}t), \end{array} $$

and using it in (20) and (21), with the help of condition (24), we write *φ*_0_ = *a*_1_ + *i* *a*_2_, *φ*_1_ = *u* + *i* *v*, *ψ*_0_ = *b*_1_ + *i* *b*_2_, *ψ*_1_ = *u*_1_ + *i* *v*_2_ where we are taking *a*_1_ = *a*_2_ for the sake of simplicity, and we separate imaginary and real parts as follows:

32 $$ P_{1}\frac{\partial^{2}u}{\partial x^{2}}-\frac{\partial v}{\partial t}+Q_{1}u+Q_{2}a_{1}\lambda_{1}=0, $$

33 $$ P_{1}\frac{\partial^{2}v}{\partial x^{2}}+\frac{\partial u}{\partial t}+Q_{1}v+Q_{2}a_{2}\lambda_{1}=0, $$

34 $$ c_{0}\frac{\partial^{2}\lambda_{1}}{\partial x^{2}}+\frac{\partial^{2} \lambda_{1}}{\partial t^{2}}+w_{g}(1-2\alpha_{0}\lambda_{0}+3\gamma_{2}{\lambda_{0}^{2}})\lambda_{1}+2c_{2}(ua_{1}+a_{2}v)=0, $$

35 $$\begin{array}{@{}rcl@{}} \frac{\partial^{2}u_{1}}{\partial t^{2}}&+&2w_{0}\frac{\partial v_{2}}{\partial t}-{w_{0}^{2}}u_{1}+c_{1}\left( \frac{\partial^{2} u_{1}}{\partial x^{2}}-2K\frac{\partial v_{2}}{\partial x}-K^{2}u_{1}\right)\\ &-&\beta((b_{1}\lambda_{1}+\lambda_{0} u_{1})-2\alpha_{0}\lambda_{0}b_{1}\lambda_{1}- 3b_{1}{\lambda_{0}^{2}}\gamma_{2}\lambda_{1}-\gamma_{2}{\lambda_{0}^{3}}u_{1})=0, \end{array} $$

36 $$\begin{array}{@{}rcl@{}} \frac{\partial^{2}v_{2}}{\partial t^{2}}&-&2w_{0}\frac{\partial u_{1}}{\partial t}-{w_{0}^{2}}v_{2}+c_{1}\left( \frac{\partial^{2} v_{2}}{\partial x^{2}}+2K\frac{\partial u_{1}}{\partial x}-K^{2}v_{2}\right)\\ &-&\beta((b_{2}\lambda_{1}+\lambda_{0} v_{2})-2\alpha_{0}\lambda_{0}b_{2}\lambda_{1}- 3b_{2}{\lambda_{0}^{2}} \gamma_{2}\lambda_{1}-\gamma_{2}{\lambda_{0}^{3}}v_{2})=0. \end{array} $$

Then, we insert

37 $$ u=u_{01}\exp i(K_{1}x-{\Omega} t)+c.c., $$

38 $$ v=v_{01}\exp i(K_{1}x-{\Omega} t)+c.c., $$

39 $$ \lambda_{1}=\lambda_{01}\exp i(K_{1} x-{\Omega} t)+c.c., $$

40 $$ u_{1}=u_{10}\exp i(K_{1} x-{\Omega} t)+c.c., $$

41 $$ v_{2}=v_{20}\exp i(K_{1} x-{\Omega} t)+c.c., $$

into (32)–(36), where *K*_1_ and Ω are the perturbation wave number and frequency, respectively. *cc* is the complex conjugate. We arrive to a five coupled linear equations

42 $$ M(u_{01},v_{01},\lambda_{01},u_{10}, v_{20})^{T}=0 $$

with

$$M=\left( \begin{array}{lllll} m_{11} & i{\Omega} & m_{13} & 0 & 0 \\ -i{\Omega} & m_{11} & m_{23} & 0 & 0 \\ m_{31} & m_{32} & -{\Omega}^{2}+m_{33} & 0 & 0 \\ 0 & 0 & m_{43} & -{\Omega}^{2}+m_{44} & -i(2w_{0}{\Omega}+m_{45})\\ 0 & 0 & m_{43} & i(2w_{0}{\Omega}+m_{45}) & -{\Omega}^{2}+m_{44} \end{array}\right) $$

where

43 $$\begin{array}{@{}rcl@{}} m_{13}=Q_{2}a_{1}, \\ m_{23}=Q_{2}a_{2}, \\ m_{32}=2c_{2}a_{2}, \\ m_{31}=2c_{2}a_{1}, \\ m_{11}=-P_{1}{K_{1}^{2}}+Q_{1}, \\ m_{45}=-c_{1}KK_{1}, \\ m_{33}=-c_{0}{K_{1}^{2}}+W_{g}(1+2\alpha_{0}\lambda_{0}+3{\lambda_{0}^{2}}\gamma_{2}), \\ m_{43}=-\beta b_{1}+2\alpha_{0}\lambda_{0}b_{2}+3{\lambda_{0}^{2}}\gamma_{2}b_{2}, \\ m_{44}=-{w_{0}^{2}}-c_{1}{K_{1}^{2}}-c_{1}K^{2}-\beta\lambda_{0}+{\beta\lambda_{0}^{2}}\gamma_{2}. \end{array} $$

The condition that (42) has a nontrivial solution requires its determinant to be zero. This gives the eigenvalue equation:

44 $$ {\Omega}^{8}-P{\Omega}^{6}-T{\Omega}^{5}+M{\Omega}^{4}+N{\Omega}^{3}+R{\Omega}^{2}+Z{\Omega}+Q=0 $$

where

45 $$\begin{array}{@{}rcl@{}} P=m_{11}^{2}+m_{33}+2m_{44}-4{w_{0}^{2}}, \\ T=4w_{0}m_{45}, \end{array} $$

46 $$ N=w_{0}(4m_{33}m_{45}-4m_{11}^{2}m_{45}), $$

47 $$ M=2m_{33}m_{44}-2m_{31}m_{11}m_{13}+m_{11}^{2}(2m_{44}+m_{33}-4{w_{0}^{2}})+m_{44}^{2}+4m_{33}{w_{0}^{2}}- m_{45}^{2}, $$

48 $$\begin{array}{@{}rcl@{}} R&=&2m_{11}^{2}m_{33}m_{44}+4m_{23}m_{44}m_{32}m_{11}-m_{33}m_{44}^{2}-(m_{11}m_{44})^{2}+8m_{11}m_{31} m_{13}{w_{0}^{2}}\\ &&-m_{33}m_{45}^{2} -(m_{11}m_{45})^{2}-4{w_{0}^{2}}m_{11}^{2}m_{33}, \end{array} $$

49 $$ Z=w_{0}m_{45}(8m_{11}m_{13}m_{31}-4m_{11}^{2}m_{33}), $$

50 $$ Q=2m_{45}^{2}m_{11}m_{13}m_{31}-m_{11}^{2}m_{33}m_{45}^{2}-2m_{11}m_{13}m_{31}m_{44}^{2}+m_{11}^{2}m_{33} m_{45}^{2}. $$

**Fig. 2:**
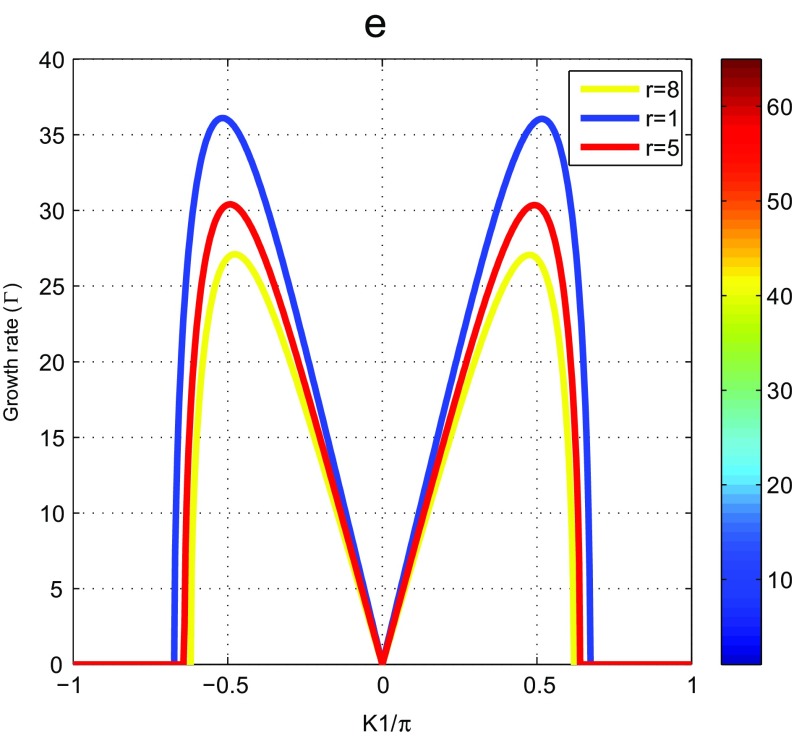
Growth rate versus the wavenumber of the perturbation *K*_1_ for *m* = 300*a* *m* *u*, *a* = 4.41Å ^−1^, *χ* = 0.3*e* *V*Å ^−1^, *D* = 0.04*e* *V*, *V* = 0.0015*e* *V*, *I* = 300*a* *m* *u* and *φ*_0_ = 10^−3^Å ^−1^

In order to solve for Ω, we need to find the roots of (44), which is a eighth order polynomial equation. It is not easy to obtain analytical results. The coefficients *P*, *T*, *M*, *N*, *R*, *Z* and *Q* add to this difficulty, because a full listing requires several pages. In this framework, we plotted in Fig. 1, these seven quantities with respect to the wave number *K*_1_, and the following results have been observed: To make sure that our system is stable we have plotted the parameters of (44), as function of wave number *K*_1_. We observe two regions. The higher zone corresponding to stability and the lower zone corresponding to instability (see Fig. 1a, b and c). One obtains the same result with Tabi et al. [24]. We also plot the growth rate of instability and one finds a similar result with that obtained by Tabi et al. [24]. But it is necessary to note here a change of concavity obtained in Fig. 1a and e due to the presence in our system of another degree of freedom. This change of concavity explains the denaturation observed in an experimental way on the dynamics of DNA molecule. All particles of the molecular chain can vibrate in opposite direction, which creates the oscillation motions of double helix. Consequently the bases pair separates completely from the double helix. The regions of instability are regions where the initial plane wave is supposed to break into trains of soliton like objects. We have plotted on the Fig. 2, the behavior of the growth rate of instability according to the size of the nucleotide. When it is high the peak of instability decreases. The nucleotide size clearly fluctuates with instability.

## Numerical experiment

**Fig. 3:**
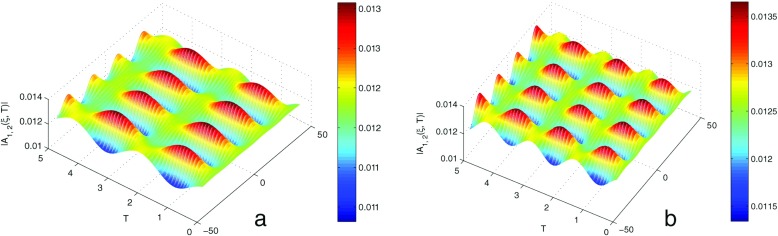
Spatiotemporal evolution of the amplitude of the initial plane wave which breaks into a wave train having the shape of a soliton-like object in DNA molecule, as predicted by the analytical predictions for *m* = 300*a* *m* *u*, *a* = 4.41Å ^−1^, *χ* = 0.3*e* *V* Å ^−1^, *D* = 0.04*e* *V*, *V* = 10^−3^ *e* *V* see Fig. 3a, and *V* = 15^−3^ *e* *V* see Fig. 3b, *r* = 3 Å ^−1^, *I* = 300*a* *m* *u* and *φ*_0_ = 10^−3^Å ^−1^

According to results obtained by many authors about the modulational instability, it is known that linear stability analysis can determine the instability field in space parameter and envisage qualitatively how the amplitude of a modulation sideband evolves at the onset of the instability. However, such an analysis is based on the linearization around the undisturbed wave carrier. Clearly, the linear approximation should fail at large time scales as the amplitude of an unstable sideband develops exponentially. Linear stability analysis therefore cannot indicate the long time evolution of a modulated nonlinear plane wave. In order to check the validity of our analytical approach and to explore the formation of localized modes, we carried out numerical simulations of our model by using the standard Fourier Transform method, with an integration time step of 0.055*s*. In our numerical simulations, the initial conditions are at time *t* = 0, are coherently modulated plane waves of the form: *λ*_*n*_ = *ψ*_*n*_ = *λ*_0_[1 + 0.01cos(*K* *n*)]cos(*K*_0_ *n*) and *φ*_*n*_ = *φ*_0_(1 + 0.01exp(*i* *K* *n*))exp(*i* *K*_0_ *n*), where *K* = *π*/65, and *K*_0_ = 3*π*/65 are the wavenumbers for the perturbation and the carrier waves , respectively. For our study, we use *λ*_0_ = *φ*_0_ = 0.002. Our aim in this numerical analysis is to point out the impact of the tight-binding hopping parameter on the occurrence of soliton-like objects induced by MI. In Fig. 3, we see that the initial condition tends to disintegrate during the propagation, leading to break-up of the wave into a pattern of wave trains. In fact, nonlinear interactions can give rise to very stable excitations, called solitons, which can travel without changing their shape. These excitations are very robust and important in the coherent transfer of energy and charges. If, in the first case, the patterns are constant, the second case shows how increasing *V* influences the distribution of wave patterns. One can conclude that MI is the most standard mechanism through which bright solitons or solitary structures can appear in physical systems. Dauxois et al. [22–29] have suggested that, such localized oscillations can be precursor of bubbles that appear in the thermal denaturation of DNA. They also showed that those structures could be used to describe the leading phenomena known as replication and transcription.

**Fig. 4:**
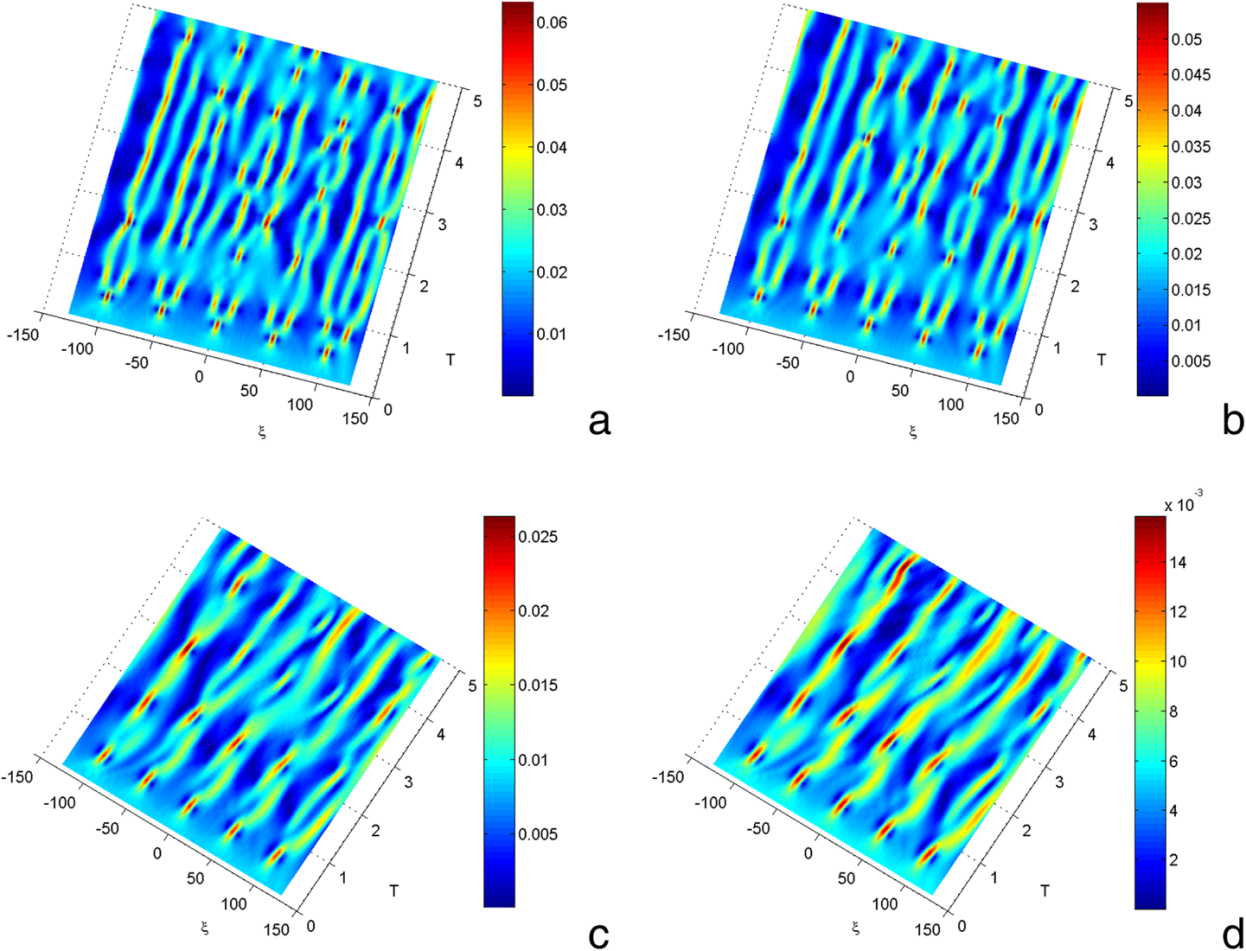
Manifestations of modulational instability of charge transport in the DNA model under the influence of charge-vibrational and rotational coupling constant for *m* = 300*a* *m* *u*, *a* = 4.41 Å ^−1^, *D* = 0.04*e* *V*, *V* = 0.1*e* *V*, *r* = 0.3Å ^−1^, *I* = 300*a* *m* *u*, *m* = 300*a* *m* *u*, *φ*_0_ = 10^−1^Å ^−1^, with **a** *χ* = 0.6*e* *V*Å ^−1^, **b** *χ* = 0.8*e* *V*Å ^−1^, **c** *χ* = 1.4*e* *V*Å ^−1^, **d** *χ* = 1.8*e* *V*Å ^−1^

We observe in Fig. 4 the formation of localized structures thus translating the emergence of charges. These charges can migrate in all the directions of the wave propagation. The charges can occupy the pairs of bases, or can meet in a site given on the molecule of DNA, and give place to a collision (Fig. 4a–b). The charges are trapped within the molecule. For *χ* = 0.6 and *χ* = 0.8 one observes an electronic distribution of charges which migrates within the molecule. But starting from *χ* = 1.6 − 1.8, this charge distribution decreases and one observes small radiations coming from the sites. The charge localizes oneself and tends to form thin rows on fewer lattices. This result was suggested by Berashevich et al. [30] which considered the Peyrard-Bishop-Holstein model in the presence of an electric field. As a whole, increasing the coupling constant of charges reduces the instability domains and better enhances spreading, in terms of charge pattern. We note that there is explosion of domains of instabilities when the value of *χ* decreases; therefore, it is possible to obtain a chaotic situation when all the molecule is saturated by charges. The charge density decreases when *χ* increases and the number of areas of instability falls when *χ* increases.

## Conclusion

We have investigated load spreading through MI in a DNA model. The linear stability analysis has shown that the model could be subjected to MI, as indicated by the representation of growth rate of instability. These analytical predictions have been verified numerically, where patterns of charge have been displayed. In this case, we have observed the region of stability/instability due to the nucleotide size. The impact of the coupling constant *χ* has been pointed out, and it has been found that increasing the value of *χ* better reduces the instability domains, and enhances charge spreading, in terms of charge pattern. We also showed the effect on size of nucleotides and the coupling transfer constant *V*. But other factors are not treated here, such as noise and thermal effect or internal vibrational excitations can also have a non-negligible influence on charge propagation.

- The authors declare that they have no competing financial interests.
- There are no competing interests related to this work.
- There are no known conflicts of interest associated with this work.
